# Impact of radiation techniques on lung toxicity in patients with mediastinal Hodgkin’s lymphoma

**DOI:** 10.1007/s00066-020-01682-0

**Published:** 2020-09-18

**Authors:** Niklas Benedikt Pepper, Michael Oertel, Christopher Kittel, Kai Jannes Kröger, Khaled Elsayad, Uwe Haverkamp, Hans Theodor Eich

**Affiliations:** Department of Radiation Oncology, University Hospital Münster, Albert-Schweitzer-Campus 1, Building A1, 48149 Münster, Germany

**Keywords:** Biological evaluation, Normal tissue complication probability, Second malignancy, IMRT, 3D-CRT

## Abstract

**Purpose:**

Mediastinal radiotherapy (RT), especially when combined with bleomycin, may result in substantial pulmonary morbidity and mortality. The use of modern RT techniques like intensity-modulated radiotherapy (IMRT) is gaining interest to spare organs at risk.

**Methods:**

We evaluated 27 patients who underwent RT for Hodgkin’s lymphoma between 2009 and 2013 at our institution. For each patient, three different treatment plans for a 30-Gy involved-field RT (IFRT) were created (anterior-posterior-posterior-anterior setup [APPA], 5‑field IMRT, and 7‑field IMRT) and analyzed concerning their inherent “normal tissue complication probability” (NTCP) for pneumonitis and secondary pulmonary malignancy.

**Results:**

The comparison of different radiation techniques showed a significant difference in favor of standard APPA (*p* < 0.01). The risk of lung toxicity was significantly higher in plans using 7‑field IMRT than in plans using 5‑field IMRT. The absolute juxtaposition showed an increase in risk for radiation pneumonitis of 1% for plans using 5‑field IMRT over APPA according to QUANTEC (Quantitative Analyses of Normal Tissue Effects in the Clinic) parameters (Burman: 0.15%) and 2.6% when using 7‑field IMRT over APPA (Burman: 0.7%) as well as 1.6% when using 7‑field IMRT over 5‑field IMRT (Burman: 0.6%). Further analysis showed an increase in risk for secondary pulmonary malignancies to be statistically significant (*p* < 0.01); mean induction probability for pulmonary malignoma was 0.1% higher in plans using 5‑field IMRT than APPA and 0.19% higher in plans using 7‑field IMRT than APPA as well as 0.09% higher in plans using 7‑field IMRT than 5‑field IMRT. During a median follow-up period of 65 months (95% confidence interval: 53.8–76.2 months), only one patient developed radiation-induced pneumonitis. No secondary pulmonary malignancies have been detected to date.

**Conclusion:**

Radiation-induced lung toxicity is rare after treatment for Hodgkin lymphoma but may be influenced significantly by the RT technique used. In this study, APPA RT plans demonstrated a decrease in potential radiation pneumonitis and pulmonary malignancies. Biological planning using NTCP may have the potential to define personalized RT strategies

## Introduction

Hodgkin’s lymphoma occurs within a relatively young mean age of onset. To ensure high rates of long-term survivors, combinations of chemotherapy and radiation treatment (RT)—and today immunotherapy and RT—have been proven effective in enhancing tumor control and overall survival for patients [1, 2]. However, the therapy itself can cause short-term complications such as pneumonitis or long-term toxicities like fibrosis or second malignancy. It can also cause significant pulmonary morbidity and mortality, specifically regarding the use of bleomycin as part of standard chemotherapy where older patients are at higher risk [3, 4].

Reducing these therapy-associated toxicities has become a crucial focus in modern lymphoma research [5–7]. Chemotherapy regimens and radiation doses have been reduced depending on the patients’ initial staging [8, 9] and treatment response [10, 11] in an effort to de-escalate harmful secondary effects. By contrast, the extent of radiation treatment dose and volume has gradually been downsized from extended field to involved field (IFRT; [12]) and subsequently to the involved site (ISRT) or even involved node radiation therapy (INRT; [13]).

The constant adjustment of treatment concepts maintains high tumor control and lowers treatment toxicity. Technical advances allow dose volumes to be distributed more conformally. At the same time, the shift from using three-dimensional conformal radiation therapy (3D-CRT) to intensity-modulated radiation therapy (IMRT) minimizes the exposure to high doses for organs at risk. Since different radiotherapeutic techniques result in different distributions of high- and low-dose volumes, the decision on which treatment is best suited remains unclear as the superiority of IMRT over standard anterior-posterior-posterior-anterior (APPA) setups has yet to be proven.

In our study, we compared radiation-associated pulmonary toxicity of different IFRT planning solutions for patients with mediastinal Hodgkin’s lymphoma. Furthermore, we analyzed biological evaluation tools to generate individual normal tissue complication probabilities (NTCP) and their potential benefits for a modern radiation treatment evaluation.

## Material and methods

Data were gathered from a pool of 27 patients treated for Hodgkin’s lymphoma in the Department for Radiation Oncology of the University Hospital of Münster between 2009 and 2013. A total of 14 male and 13 female patients between 15 and 80 years old were included. Criteria for selection were patients with thoracic manifestation whose lungs were organs at risk for treatment planning. Cases varied in stages (with 22 being in Ann Arbor stage II, two patients in stage I, and three patients in stage IV) and, therefore, in clinically applied treatment strategies.

In order to achieve comparability, three treatment plans for 30.6-Gy IFRT of mediastinal lymphoma manifestation were calculated for each patient. Treatment planning was realized based on a computed tomography (CT) scan with i.v. contrast enhancement, using the Varian Eclipse Version 10.0 (provided by Varian Medical Systems located in Palo Alto, CA, USA) for contouring target volumes and organs at risk. The definition of planning target volume (PTV) followed the contouring guidelines for IFRT by the International Lymphoma Radiation Oncology Group (ILROG) [13, 14] with each separate plan applying 30.6 Gy in 17 fractions of 1.8 Gy five times a week.

**Fig. 1:**
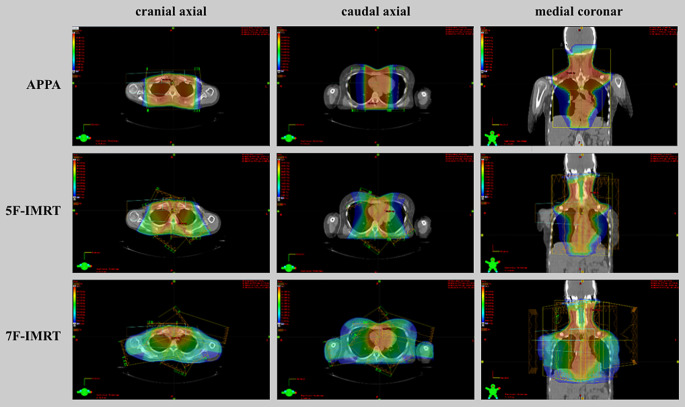
Example for different dose distributions in APPA, 5‑field intensity-modulated radiotherapy (*5F-IMRT*), and 5‑field intensity-modulated radiotherapy (*7F-IMRT*)

Physical realization of these requirements was achieved in three alternative plans for every case: one using a standard 3D-conformal setup of APPA radiation and two using IMRT solutions, one using five fields (5F-IMRT) and one using seven fields (7F-IMRT). Figure 1 illustrates the dose distributions of these three methods in one exemplary case.

In the planning process, a dose grid of 2.5 mm and the dose calculation algorithm AAA were used, IMRT was performed with fixed-beam angles. For the 5‑field IMRT beam, angles of 20°, 145°, 180°, 215°, and 340° were used. The 7‑field IMRT was equally distributed in 51° steps (0° up to 306°). The Eclipse planning system generates optimal fluences in the optimization process by adjusting the ray weights using a gradient optimization method. The optimal fluences represent the ideal field modulation in which the physical and mechanical characteristics of the MLC device have not been taken into account. The real fluence is achieved by the Leaf Motion Calculator after optimization. Initial dose objectives were set to values based on QUANTEC (Quantitative Analyses of Normal Tissue Effects in the Clinic) data [17], followed by patient-individual optimization to achieve ideal treatment plans, which conformed to the ILROG guidelines for dose constraints in Hodgkin’s lymphoma therapy dosimetrically [13]. Plan normalization to PTV-Dmean = 100% of the prescribed isodose was performed to create identical dose setups. After optimization to ensure clinical applicability, dose–volume histogram (DVH) data were gathered as an indication of the difference in dose distributions of the particular methods. Each treatment plan was assessed via the tool for biological evaluation implemented in Varian Eclipse and by using the Lyman–Kutscher–Burman model for modelling biological response probabilities of different tissue [15], predicting normal tissue complication probabilities (NTCPs) as well as tumor control rates (TCPs).

In accordance with this model, both lungs were selected as organs of interest. The risk calculation of the endpoint was set to the mildest selectable form of pulmonary toxicity, i.e., “symptomatic or radiographic pneumonitis,” which was evaluated for each treatment plan. Furthermore, two sets of parameters were used as a basis for the calculation of the NTCPs: first, the parameters suggested by Burman [16], which are the standard setup when using Varian Eclipse’s biological treatment evaluation. Second, the parameters published by QUANTEC [17], which aim at bringing model predictions closer to real clinical outcomes.

Adjustable parameters of the model were *n* and *m* as parameters representing steepness, TD50 for positioning, and alpha/beta for the curvature of the curve modeling the dose–response of the selected tissue. In order to include long-term toxicity, the induction risk of radiation-associated second malignancies in each plan was assessed using the function developed by Schneider et al. [18]. This function is based on organ-equivalent dose and models dose–response by utilizing several patient-, tissue-, and plan-specific parameters to predict the risk of malignancies.

All data were statistically analyzed to evaluate the potential significance of correlations between the treatment planning modality (APPA vs. 5‑field IMRT vs. 7‑field IMRT) and associated NTCP as well as second malignancy probability, using the Shapiro–Wilk test and the Kolmogorov–Smirnov test for distribution analysis, and the Wilcoxon rank test to check for significance. The overall accuracy of these theoretical or predicted risks, respectively, was determined by comparing them with each patient’s clinical follow-up through the examination of medical aftercare reports and radiographic follow-ups.

## Results

The comparison of DVH data of individual treatment plans showed a shift in the balance of tissue affection with low as well as high doses when switching from 3D-CRT to IMRT: Volumes irradiated with low doses from 5 to 15 Gy (V5–V15) were more prominent in median and mean when using IMRT (5-field as well as 7‑field) compared with APPA. Mean and median volumes with doses between 20 and 30 Gy (V20–V30) in the APPA plans exceeded those in the IMRT plans. Comparing IMRT plans, 5‑field IMRT showed dose distributions very similar to 7‑field IMRT, but with overall smaller low-dose areas. Additional analysis of the DVH showed that the volumes being affected by a specific dose when using IMRT over APPA were larger up to a dose of 19.3 Gy (being 63% of the target dose of 30.6 Gy). Therefore, IMRT showed more tissue affected with low-dose areas, whereas APPA had smaller low-dose areas but larger high-dose areas.

Using the biological evaluation tool to investigate suspected differences in normal tissue complication probability between these dose distributions, and analysis using the standard parameters by Burman as well as the new QUANTEC parameters, yielded the results shown in Table 1.

**Table 1:** Normal tissue complication probabilities associated with different planning methods

	Plan	NTCP Burman left lung [%]	NTCP Burman right lung [%]	NTCP QUANTEC left lung [%]	NTCP QUANTEC right lung [%]
Median	APPA	0.02	0.01	5.07	4.64
	5F-IMRT	0.07	0.03	6.35	5.75
	7F-IMRT	0.1	0.16	7.28	7.96
Mean	APPA	0.26	0.23	5.86	5.41
	5F-IMRT	0.44	0.35	6.97	6.35
	7F-IMRT	0.94	0.98	8.36	8.18
Range min.	APPA	0	0	1.39	2.34
	5F-IMRT	0	0	1.62	2.39
	7F-IMRT	0	0	1.74	2.34
Range max.	APPA	2.07	2.37	12.24	12.55
	5F-IMRT	3.97	2.9	15.03	13.90
	7F-IMRT	5.12	7.93	16.38	18.16

The analysis demonstrated noticeably higher values using the QUANTEC parameters; NTCPs clearly tended to be higher in 5‑field IMRT than in APPA and the highest in 7‑field IMRT in both analyses. The following statistic evaluation showed the differences to be highly significant with values of *p*<0.01 for the differences between APPA and 5‑field IMRT, APPA and 7‑field IMRT, as well as 5‑field and 7‑field IMRT.

In the next step, the inherent risks of treatment plans were analyzed by employing the formula by Schneider et al. with the following objectives. First, scrutinizing the high-grade toxicity of pulmonary second malignoma. Second, comparing the risks associated with different plans. The risks were calculated as lifetime risks based on the statistical life expectancy of each patient without taking into account their respective medical history. The results are shown in Table 2.

**Table 2:** Second malignoma probability associated with different planning methods

	APPA [%]	5F-IMRT [%]	7F-IMRT [%]
Median	0.35	0.44	0.53
Mean	0.37	0.47	0.56
Range min.	0.06	0.08	0.1
Range max.	1.27	1.62	1.85

The APPA plans were associated with a mean lifetime risk of 0.37% (with a vast range between 0.06 and 1.27%), 5‑field IMRT with a mean risk of 0.47% (range: 0.08–1.62%) and 7‑field IMRT with a mean risk of 0.56% (range: 0.1–1.85%). Again, 3D conformal beam setup demonstrated a significantly lower risk (*p* < 0.01) for pulmonary toxicity compared with IMRT (with 5‑field IMRT bearing a statistically significant lower risk for second malignoma than 7‑field IMRT as well, i.e., *p* < 0.01).

Direct comparison of planning methods led to the following results: Choosing 5‑field IMRT over APPA increased the risk for pulmonary second malignancy by a mean 0.1%; i.e., a relative increase of 27%. Choosing 7‑field IMRT over APPA increased the risk by a mean of 0.19% (relative increase of 51%) and choosing 7‑field over 5‑field IMRT increased the risk by a mean 0.09% (relative increase of 19%).

Overall, 20 of the patients included were treated with APPA plans, four were treated with 5‑field IMRT and three with 7‑field IMRT. When assessing follow-up data to check for validity of the theoretical risks, one patient reported prolonged symptoms of pulmonary toxicity (shortness of breath 6 months after end of RT via 7F-IMRT) without detectable limitations in lung capacity. One follow-up thoracic CT scan showed signs of potential radiation pneumonitis in a low-dose area; the patient neither reported any symptoms nor could any limitations in lung capacity be detected within 6 months of APPA-RT.

Therefore, the reported toxicities drawn from patient aftercare reports as well as radiographic follow-up fit the overall low probabilities of pulmonary toxicity shown in the analysis. No patient developed pulmonary malignancy. Mean follow-up time was 65 months (95% confidence interval: 53.8–76.2 months), with seven patients being lost to follow-up before the benchmark of 60 months.

## Discussion

The aim of this study was to evaluate whether the choice of IMRT over classic 3D-CRT benefits patients undergoing treatment for mediastinal Hodgkin’s lymphoma regarding the minimization of lung toxicity. It must be stated that the analyzed cohort was irradiated with IFRT, which is no longer the standard in target volume definition for lymphoma patients and has since been succeeded by ISRT or INRT. This de-escalation in radiotherapy volume has proven to be equally beneficial for tumor control [19] and is currently reviewed further in the German HD17 trial [20]. Nevertheless, this study focused on IFRT because of its long history of application. This amounts to more clinical data being available due to longer periods of patient follow-up.

Comparing the DVHs of the analyzed treatment plans demonstrated significant differences among the various modalities in terms of dose distribution in organs at risk. Subsequently, variations in possible treatment toxicities were proven to be statistically significant: In line with other studies, IMRT has proven to be associated with a higher risk of pulmonary toxicity than 3D-CRT [21]. Other studies point out that this increase of low-dose tissue irradiation might be compensated by superior target volume coverage delivered by IMRT [22, 23]. The additional scientific effort has since shown other treatment modalities to effectively achieve more efficient protection of normal tissue: Both proton therapy and VMAT may be beneficial [22, 24] while deep inspiration breath-hold serves as a strategy to reduce treatment toxicity over multiple modalities [22].

With a wide array of possible strategies, finding the optimal treatment planning method for mediastinal radiation requires a multifaceted approach. This study has scrutinized lung toxicity as a surrogate organ at risk because it can be detected easily during follow-up. It has also been chosen because of the variety in possible toxicity, ranging from short-term and relatively mild (i.e., radiation pneumonitis) to long-term and severe (i.e., pulmonary second malignancy) symptoms. These endpoints were not only already implemented in modules for biological evaluation in standard treatment planning software but also modeled in dose–response equations. If proven reliable, they could be used in clinical routine.

The overall values for pulmonary toxicity for patients with mediastinal Hodgkin’s lymphoma have been determined as marginal, a finding that aligns with other studies showing similar results [21, 25, 26]. Nevertheless, the focus on lung toxicity represents only a fraction of information when analyzing possible treatment toxicity. As studies have shown, other thoracic organs such as the heart and the mammary glands also demand a high degree of caution when evaluating treatment plans; exposing them to radiation may lead to severe late treatment toxicity [27].

Mediastinal radiation bears the risk of second malignancy [21, 28]. It is, therefore, crucial to render it an important focus point in treatment evaluation, especially when treating a disease with a relatively young mean age of onset. For example, radiation to the mammary glands has proven to be a relevant but reducible risk factor for developing breast cancer [24, 29, 30].

In this analysis, possibilities for lifetime risks of pulmonary second malignancy were shown to be wide-ranging—a result of the high variation in the age of onset and life expectancy, respectively. Nevertheless, this statistical analysis is proof that the correlation of a higher risk of pulmonary malignancy with IMRT planning vs. APPA planning is statistically significant. It demonstrates that modern solutions such as IMRT do not necessarily result in lesser toxicity, at least concerning the lungs. Since this evaluation focused only on pulmonary malignoma, further research concerning other organs at risk (especially regarding the mammary glands) is necessary. The fact that no pulmonary malignancies were detected in the patients’ history after initial treatment should not be overestimated since the duration of follow-up is not long enough to present valid data.

Regarding the calculated differences, it is furthermore important not to over-scrutinize relative differences when comparing different planning techniques: Big relative increases in risk (e.g., the relative increase in risk shown for pulmonary malignancies of 27 and 51% when choosing 5‑field or 7‑field IMRT over APPA) can accompany marginal absolute differences and should therefore not be overvalued when it comes to clinical relevance.

Since the complexity of circumstances regarding advantages and disadvantages of every modality does not allow for a universal solution, treatment individualization is the most feasible way to further decrease treatment toxicity. The NTCP values are an easily accessible source of additional information when comparing treatment plans and allows for a more informed decision on a specific treatment plan. Biological evaluation offers the possibility to individually adjust radiation treatment to a patient’s risk profile, specifically regarding preexisting conditions and previous treatment modalities.

Additional radiation-associated lung toxicity might be a key risk factor for patients who have been treated with pneumotoxic substances like bleomycin, which is commonly administered when treating Hodgkin’s lymphoma, but has been proven to increase pulmonary morbidity [3]. In a similar manner, the treatment with immune-modulatory checkpoint inhibitors such as nivolumab potentially increases the probability of pulmonary complication [31]. With more evidence supporting this treatment as a valid new option [1], this study’s focus on minimizing radiation-associated adverse events gains further importance.

This study has shown that choosing 3D-CRT enables a reduction of potential lung toxicity, making it a valuable option when treating mediastinal Hodgkin’s lymphoma. Taking the previously discussed results of other researchers into consideration, it is obvious that further reductions in radiation treatment toxicity are achievable but complex. Methods of biological evaluation can be useful to assess the advantages and disadvantages of treatment plans but must be considered individually for each case. Our study shows limitations in terms of the relatively short periods of follow-up regarding secondary neoplasia, which typically occur after long periods. Further limitations are related to the focus on IFRT, which is no longer the standard treatment for Hodgkin’s lymphoma.

## Conclusion

The occurrence of clinically relevant radiation-induced lung toxicity is relatively rare for patients with Hodgkin’s lymphoma. Nonetheless, it can impair treatment outcomes and increase complication probabilities in other organs at risks, such as the heart or the mammary glands. This study shows that using APPA treatment plans over IMRT may reduce the risk of pneumonitis as well as pulmonary second malignancy. Therefore, the choice of a specific treatment modality impacts treatment outcome and must be evaluated for each case individually. Especially patients with preexisting conditions affecting the lung (e.g., chronic obstructive pulmonary disease, severe asthma, a history of lung injury due to nicotine dependence, Covid-19 or other diseases) might benefit from a closer evaluation of different treatment methods in this regard. The use of biological evaluation might be a feasible option to further improve patient outcome.
